# Supplementary material for: The Role of Copper in the Regulation of Ferroportin Expression in Macrophages
Source: Cells. 2021 Aug 31;10(9):2259. doi: 10.3390/cells10092259 (PMC8469096; doi:10.3390/cells10092259)
Supplement: Supplementary file 1 [file cells-10-02259-s001.zip › cells-1346630-supplementary0827/Table S2.pdf]

Table S2. List of antibodies for Western blot

Target protein	Primary Ab	Dilution	Secondary Ab	Dilution
**Fpn**	Affinity purified rabbit anti-mouse Fpn, kind gift from *F.Canonne-Hergaux*, France	1:500	Goat anti-rabbit, polyclonal, Cell Signaling Technology Cat# 7074,	1:10,000
**H-Ft**	Rabbit polyclonal, Abcam, Cat# ab65080	1:1,000	Goat anti-rabbit, polyclonal, Sigma-Aldrich Cat# A6154	1:10,000
**L-Ft**	Rabbit polyclonal, Abcam, Cat# ab69090	1:1,000	Goat anti-rabbit, polyclonal, Sigma-Aldrich Cat# A6154	1:10,000
**β-actin**	Rabbit monoclonal, Thermo Fisher Scientific, Cat# MA5-11869	1:1,000	Goat anti-rabbit, polyclonal, Sigma-Aldrich Cat# A6154	1:10,000
**Flotilin-2**	Mouse monoclonal, Santa Cruz Biotechnology Cat# 28320	1:500	Goat anti-mouse, polyclonal, Sigma-Aldrich Cat# A5278	1:10,000
**TfR1**	Mouse monoclonal, Thermo Fisher Scientific, Cat# 13-6800	1:1,000	Goat anti-mouse, polyclonal, Sigma-Aldrich Cat# A5278	1:10,000
