# Supplementary material for: The Role of Copper in the Regulation of Ferroportin Expression in Macrophages
Source: Cells. 2021 Aug 31;10(9):2259. doi: 10.3390/cells10092259 (PMC8469096; doi:10.3390/cells10092259)
Supplement: Supplementary file 1 [file cells-10-02259-s001.zip › cells-1346630-supplementary0827/Table_S1.pdf]

Table S1. List of primers for qPCR

Gene	Primer Sequence 5′–3′	
	**Forward**	**Reverse**
Rpl 4	TCCGAGCACCACGCAAGAAG	TCTGGCCTGGCGAAGAATGG
Rpl 19	CAAGCTGAAGGCAGACAAGGC	CGCTTTCGTGCTTCCTTGGT
Fpn	GCCAGTGTCCCCAACTACCA	ACCAAAGACCGATTCTAGCAGCA
Hprt	AGGGAGAGCGTTGGGCTTAC	TCGCTAATCACGACGCTGGG
Nqo1	CTGGCCGATTCAGAGTGGCA	GAGTGGGGTCTCCTCCCAGA
Ctr1	GGGGCTTACCCTGTGAAGACTTT	GGTGGTGAGGTGGCATGGTA
Atp7a	TTCCCATCGCTGCTGGAGTTT	GCTCCGGGGATGCAACTCATA
TfR1	TCGCTTATATTGGGCAGACC	CCATGTTTTGACCAATGCTG
Hamp	TCTCCTGCTTCTCCTCCTTG	CAATGTCTGCCCTGCTTTCT
Lip5	ACAGCTTCCCAGGGGGATA	ACTGGCTTCCGTGAACAGAG
Chmp5	TACGGCACCCCAGAGTTAGA	CAGCACGCCATCCTTGTTTT
Psma1	TGACGATCACCGAACCGTAG	GCTCTGACTGTGCTCTCTTCA
Psmc4	GATGCTCACCTCAGACCAGA	TCGAAGTGTGTAAGCGGGAG
Usp14	CCATGCCACTCTACTCTGTTAC	GCATCTGCTGACCCCATCATT
Ubc	CCACACAAAGCCCCTCAATC	AAAGATCTGCATCGTCTCTCTC
